# Development of Glutathione Hydrogel Carriers Containing Zinc Oxide Microparticles for Skin Regeneration Processes

**DOI:** 10.3390/ijms26041395

**Published:** 2025-02-07

**Authors:** Dominika Träger, Katarzyna Młyniec, Katarzyna Haraźna, Dagmara Słota, Karina Niziołek, Josef Jampilek, Agnieszka Sobczak-Kupiec

**Affiliations:** 1 Department of Materials Science, Faculty of Materials Engineering and Physics, Cracow University of Technology, 37 Jana Pawła II Av., 31-864 Krakow, Poland; dominika.trager@student.pk.edu.pl (D.T.); katarzyna.mlyniec@student.pk.edu.pl (K.M.);; 2 Department of Materials Science, Faculty of Materials Engineering and Physics, CUT Doctoral School, Cracow University of Technology, 37 Jana Pawła II Av., 31-864 Krakow, Poland; 3 Department of Analytical Chemistry, Faculty of Natural Sciences, Comenius University, Ilkovicova 6, 842 15 Bratislava, Slovakia; 4 Department of Chemical Biology, Faculty of Science, Palacky University Olomouc, Slechtitelu 27, 779 00 Olomouc, Czech Republic

**Keywords:** active substance carrier, zinc oxide microparticles, polyethylene glycol, sodium alginate, acne vulgaris, hydrogels

## Abstract

Skin represents the largest organ in the human body, functioning as a protective barrier against environmental factors while playing a critical role in thermoregulation. Acne vulgaris is recognized as the most common dermatological condition affecting adolescents, and if left untreated, it can result in lasting skin damage and associated psychosocial challenges. This study aims to develop innovative polymeric biomaterials that could effectively support the treatment of acne vulgaris. The synthesis of these biomaterials involves the use of polyethylene glycol 6000, sodium alginate, and the antioxidant protein glutathione (GHS) to create polymeric hydrogels. These hydrogels were generated via a UV-mediated crosslinking process. To enhance the functional properties of the hydrogels, zinc oxide microparticles (ZnO), synthesized through a wet precipitation method, were incorporated into the formulations. Characterization of the ZnO was performed using Fourier-Transform Infrared Spectroscopy (FTIR), X-ray Diffraction (XRD), particle sizer analysis, and Scanning Electron Microscopy (SEM). Additionally, the bioactivity of the synthesized materials was evaluated through incubation in media simulating physiological body fluids. The cytotoxic effects of the biomaterials were assessed using an indirect test on mouse fibroblast (L929) cells, in accordance with ISO 10993-5 guidelines. The results of our research indicate that the developed biomaterials exhibit potential as a carrier for active substances, contributing positively to the treatment of acne vulgaris and potentially improving overall skin health.

## 1. Introduction

Acne vulgaris is a chronic inflammatory skin disease that affects approximately 85% of adolescents and a significant proportion of adults, causing mild skin changes and severe scarring if not treated properly. This condition has a complex etiology involving excessive sebum production, hyperkeratinization of hair follicles, and promoting bacterial growth, resulting in skin inflammation [1,2]. Key bacterial contributors include *Cutibacterium acnes* and *Staphylococcus aureus*, which, under favorable conditions, can proliferate excessively, disrupting the balance of the skin’s microenvironment [3]. An important factor in maintaining a healthy skin barrier is its pH, which typically ranges from 4.5 to 5.5, providing an acidic environment that limits bacterial growth and supports the skin’s natural defenses. However, in acne-prone or diseased skin, the pH often shifts toward alkaline values, creating a more favorable environment for bacterial proliferation and disrupting the skin’s protective barrier. This pH imbalance exacerbates the inflammatory processes driven by enzymes and pro-inflammatory cytokines produced by these bacteria, leading to acne lesions and long-term scarring [4,5].

Oxidative stress plays a significant role in the pathogenesis of acne. It is a process resulting from the excessive production of free radicals, which are generated by various factors such as UV radiation, environmental pollution, an unhealthy diet, and stress. Free radicals interact with lipids, proteins, and DNA, causing cellular damage and disrupting their function, which leads to intensified inflammatory states and an imbalance in the skin. Excess reactive oxygen species (ROS) damage keratinocytes and fibroblasts, amplifying inflammatory processes [6]. Furthermore, ROS stimulate the immune system, enhancing its ability to defend against pathogens [7]. Consequently, elevated oxidative stress levels intensify acne damage and accelerate skin aging [8,9]. To mitigate the effects of oxidative stress, cosmetic applications such as antioxidant-rich creams and masks have been developed. These products often contain ingredients like vitamin C, vitamin E, coenzyme Q10, and botanical extracts (e.g., green tea or resveratrol) that neutralize free radicals, reduce inflammation, and support skin repair. Regular use of such formulations not only helps protect the skin from environmental damage but also promotes a balanced, healthier complexion. Understanding these mechanisms is essential for developing effective acne treatment methods [10].

Hydrogels are three-dimensional polymeric networks that exhibit remarkable elasticity and biocompatibility due to their ability to absorb large amounts of water [11]. These properties make the ideal materials for use in skincare and regenerative medicine [12]. In dermatology, hydrogels act as carriers for active substances, such as antioxidants, antibacterial agents, and anti-inflammatory compounds. Their structure allows for the controlled release of therapeutic components, enhancing treatment effectiveness. Hydrogel masks, a popular application of hydrogels, gradually deliver active ingredients while intensively moisturizing the skin [13]. According to Grand View Research, the global hydrogel mask market is rapidly growing, expected to exceed USD 200 million by 2030 [14]. Masks such as BioCellulose^®^ and Dr. Jart+ are examples of innovative hydrogel technologies that improve moisture retention and active ingredient delivery efficiency [15]. Concurrently, scientific research focuses on developing new formulations, such as hydrogels enriched with antioxidants and nanoparticles, which significantly enhance their therapeutic properties [16].

Polyethylene glycol 6000 (PEG 6000) is a hydrophilic synthetic polymer widely used in biomaterials due to its moisturizing and stabilizing properties [17]. Produced through the polymerization of ethylene oxide, PEG (6000) finds applications in various cosmetic, dermatological, and medical products [18]. In hydrogels, it serves as a crucial carrier for active substances, enabling their controlled release to the skin [19]. Its ability to retain large amounts of water supports intensive skin hydration, which is particularly important in acne treatment [20].

Sodium alginate (SA) is a polysaccharide derived from brown algae, such as *Laminaria* and *Macrocystis pyrifera*, which can form a flexible gel [21]. The production process of SA involves extraction from seaweed and neutralization with hydrochloric acid, followed by purification and drying [22]. Its chemical structure, consisting of mannuronic and guluronic acid residues, allows the formation of gels with varying stiffness and elasticity, depending on the ratio of these units [23,24]. Alginates are non-cytotoxic and are used in wound dressing construction to support tissue healing and regeneration. Additionally, they stabilize various formulations, ensuring consistency and structure in cosmetic and pharmaceutical applications [25,26].

Glutathione (GSH) is a natural tripeptide that protects cells from oxidative stress [27]. It comprises three amino acids: glutamine, cysteine, and glycine, which give it unique antioxidant properties. Naturally present in almost all cells, GSH is most concentrated in the liver, which plays a crucial role in detoxification processes [28]. In cosmetics, GSH is used in anti-aging and skin-brightening products because it inhibits melanin production, reducing skin curation [29]. Incorporating GSH into hydrogel masks enhances its stability and therapeutic effectiveness, allowing for prolonged protective and regenerative effects [30]. Moreover, to its cosmetic applications, glutathione plays a pivotal role in skin health through its association with glutathione peroxidase (GPx). GPx reduces oxidative damage caused by reactive oxygen species (ROS), which are known to exacerbate inflammation in acne vulgaris. Studies have shown that reduced GPx activity correlates with increased severity of acne, as oxidative stress disrupts sebaceous gland function and promotes inflammation. Furthermore, biogenic amines, such as dopamine and noradrenaline, influence sebaceous gland activity and inflammatory responses. Elevated levels of these amines are linked to acne severity, highlighting their contribution to disease progression. Addressing oxidative stress and biogenic amine dysregulation could improve acne management and reduce inflammation [31].

Zinc oxide (ZnO) micro-/nanoparticles have broad applications in biomaterials due to their antibacterial, anti-inflammatory, and regenerative properties [32]. Its ability to inhibit the growth of bacteria such as *Staphylococcus aureus* and *Escherichia* coli is particularly valuable in the context of rising antibiotic resistance, as these bacteria do not develop resistance to ZnO’s mechanisms of action [33,34]. This makes ZnO nano-/microparticles a promising alternative in addressing the challenges of antibiotic-resistant infections. ZnO nano-/microparticles are utilized in wound dressings, bone implants, and materials aiding skin regeneration, where they reduce inflammation, promote tissue renewal, and provide a safe and effective solution to combat resistant pathogens [35]. Its unique properties ensure its place as a key component in regenerative medicine [36].

The work presented here aimed to confirm the feasibility of obtaining bioactive polymeric masks and explore the ZnO microparticles’ synthesis conditions. The final polymeric hydrogels, intended for use in facial masks designed to treat acne vulgaris, were created using polyethylene glycol, SA, ZnO microparticles, and GSH. We conducted physicochemical and morphological analyses, as well as incubation studies in solutions that simulate body fluids for the materials obtained. Moreover, we assessed the cytotoxicity of the polymeric hydrogels enriched with GSH and ZnO microparticles using an indirect method based on the guidelines established in ISO 10993-5 [37].

## 2. Results and Discussion

### 2.1. Particle Size Measurements of Synthesized ZnO

**Figure 1:**
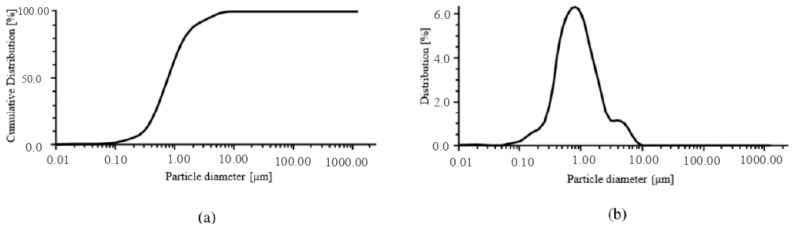
Distribution of ZnO particle sizes: (**a**) cumulative distribution of volume shares showcases the overall trend, while (**b**) volume share distribution provides a detailed breakdown of size metrics.

The measurements were conducted to assess the specific particle size distribution of ZnO, with the findings illustrated in Figure 1. The particle diameters below which 10% (D_10_), 50% (D_50_), and 90% (D_90_) of the total volume are contained, and the mean diameter (D_mean_), were determined from the correlated average particle size distribution, which is summarized in Table 1. The D_mean_ of the ZnO particle size was 2.371 μm. The particle distribution is relatively variable, demonstrated by a spread between D_10_ and D_90_, which may lead to the formation of agglomeration in the biomaterial. Taking the above into account, the alginate, a natural stabilizer, was used to synthesize the hydrogel to improve the homogeneity of the dispersion [38]. Moreover, solution sonification was performed before cross-linking the matrix to break up possible ZnO agglomerations. Notably, alginate can also be used for the in situ growth of micro/nano-particles. This is possible because the alginate structure contains ion exchange sites, which are used for nucleation as well as the subsequent growth of crystallites. This makes it possible to obtain mixtures characterized by improved dispersibility [39].

### 2.2. X-Ray Diffraction Analysis

**Figure 2:**
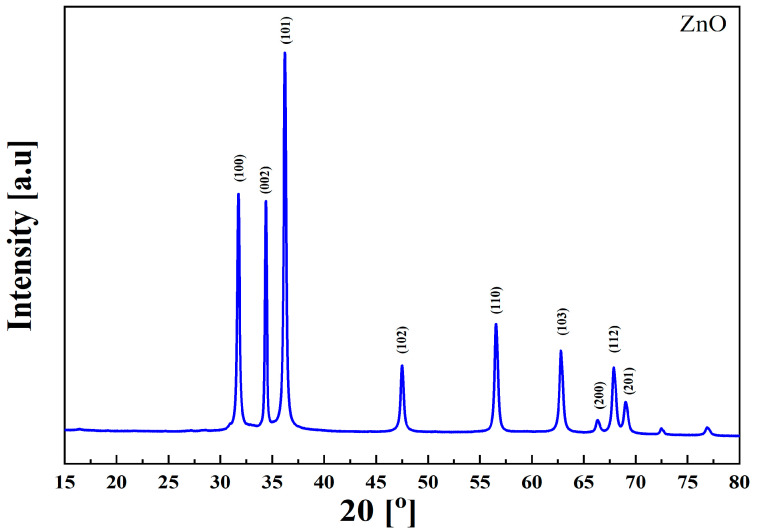
XRD diffractogram of ZnO microparticles.

ZnO nano-/microparticles are remarkable materials that have been synthesized in an impressive array of forms, including nanoflowers, nanorods, nanowhiskers, nanobelts, nanotubes, nanorings, and nanocolumns. The diverse range of synthesis techniques, such as the sol–gel method, wet precipitation, spray pyrolysis, hydrothermal processes, microwave-assisted techniques, and many others, further highlight their versatility. This variety underscores the potential applications of ZnO nano-/microparticles [40]. X-ray diffraction (XRD) analysis was carried out to determine the different phases of the synthesized ZnO microparticles. The results of the study in the form of a diffractogram showing the ZnO microparticles phases obtained are shown in Figure 2.The diffractogram indicated a high proportion of crystalline phase, which was denoted by sharp reflections at 2θ = 31.71°; 34.30°; 36.20°; 47.50°; 56.56°; 62.70°; 66.27°; 68.00°; 69.00°, corresponding to crystalline phase: 100, 002, 101, 102, 110, 103, 200, 112, and 201, respectively. Similar Results were observed by Castro-Mayorga et al. and Mendes et al. [41,42].

### 2.3. Fourier-Transform Infrared Analysis

**Figure 3:**
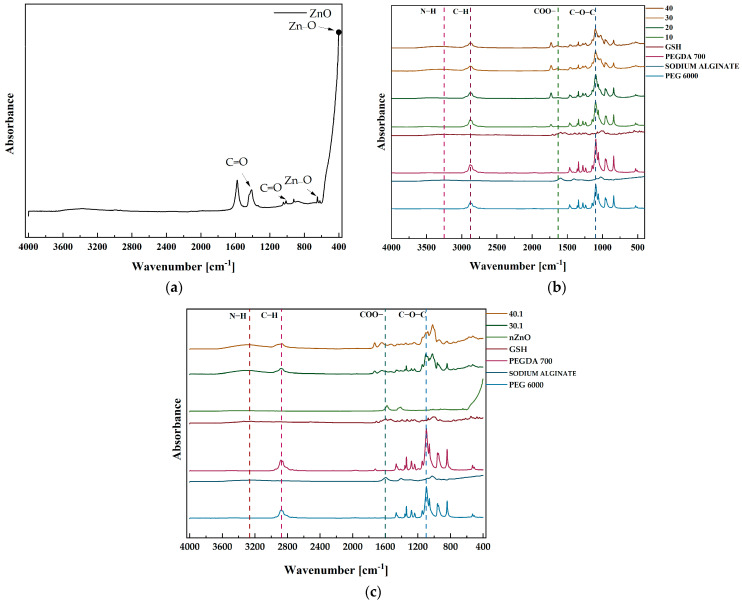
FT-IR spectra of (**a**) synthesized ZnO microparticles, (**b**) polymeric hydrogel samples, and (**c**) hydrogel counterparts containing ZnO microparticles.

Fourier-Transform Infrared Analysis (FTIR) was carried out to assess the chemical structure of the materials obtained. In our work, ZnO microparticles were produced by wet precipitation, and their spectrum is shown in Figure 3a. Confirmation of the ZnO structure was obtained by recording characteristic absorption bands in the fingerprint region. These were Zn-O stretching absorption bands from 400 to 700 cm^−1^ [43]. Moreover, the spectrum of ZnO microparticles is also characterized by absorption bands, which are responsible for asymmetric and symmetric stretching vibrations of the C=O groups. Their presence may be related to the zinc acetate synthesizing ZnO microparticles. Similar conclusions were made in the paper published by Khan et al., where ZnO nanoparticles obtained by the sol–gel method were characterized [40].

In the next step, we analyzed the chemical structure of hydrogel materials prepared from SA, PEG 6000, and PEGDA 700, with the addition of substances such as synthesized ZnO microparticles and GSH (Figure 3b,c). Characteristic absorption bands corresponding to the compounds used in the syntheses were observed on the spectra. All spectra displayed a peak at 1100 cm^−1^ , which corresponds to the vibrations of the C–O–C bond found in PEG 6000 [44]. In addition, all materials showed a peak of 2876 cm^−1^, which belongs to the C–H group vibrations and originated from PEGDA 700 [45]. Furthermore, signals corresponding to COO^−^ stretching vibrations originating from SA around 1636 cm^−1^ were also observed on all polymeric hydrogel spectra, including those containing ZnO microparticles [46]. An analogous situation occurred in the case of GSH, where vibrations of N–H groups were located in the wavenumber range of 3200–3500 cm^−1^ [47]. Importantly, no absorption bands corresponding to ZnO-derived functional group vibrations were identified in the spectra of polymeric hydrogels containing ZnO microparticles (Figure 3c). This is due to the fact that a small amount of it was introduced, compared to the rest of the polymeric components.

### 2.4. Morphology Analysis

**Figure 4:**
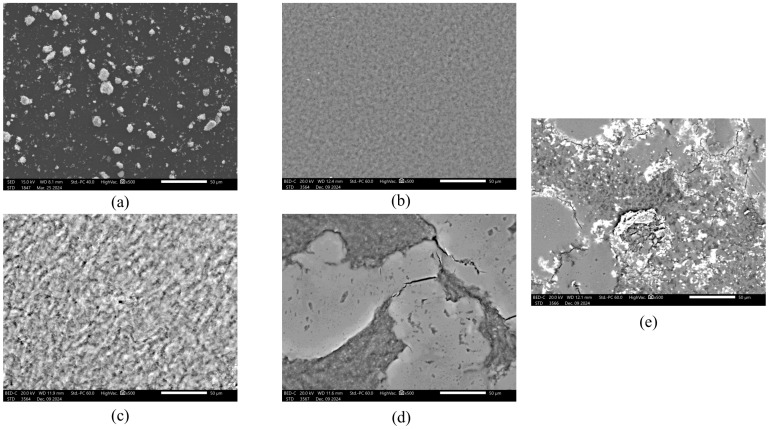
Analysis of surface morphology of (**a**) pure ZnO, (**b**) sample no. 10, (**c**) sample no. 20, (**d**) sample no. 40, (**e**) sample no. 40.1.

We assessed the homogeneity and morphology of the prepared ZnO microparticles as well as polymeric hydrogels using scanning electron microscopy (SEM). It can be observed in Figure 4a that the obtained ZnO microparticles have a tendency to agglomerate. A comparison of the polymer matrices without GSH and ZnO microparticles (Figure 4b,c) and those obtained by introducing GSH (Figure 4d) shows that with the incorporation of GSH, the surface of the materials becomes less homogeneous, with a tendency to crack. Figure 4e shows the morphology of sample 40.1. obtained by introducing the highest concentration of GSH and ZnO microparticles. In the microphotographs, both cracks and lack of homogeneity were observed, which was related to the introduction of GSH, and agglomerates of ZnO microparticles were also visible.

Along with the morphology study, EDS microanalysis was also performed. Its focus was identifying the elements and determining their abundance in the coating. The exact results of the microanalysis are shown in Table 2.

**Figure 5:**
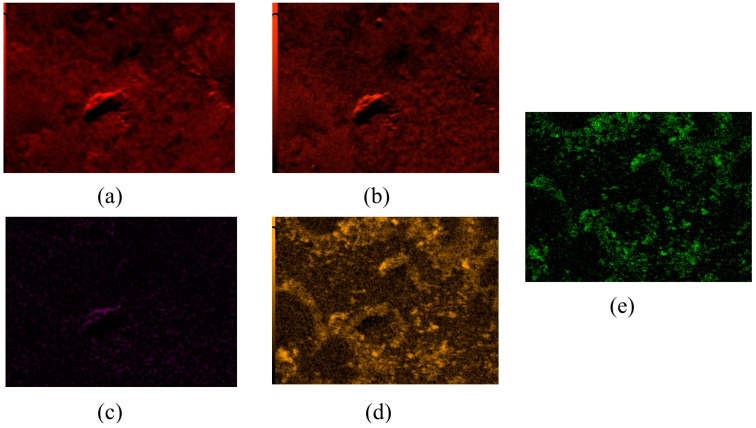
Elemental EDS microanalysis and determination of elements on the surface of material 40.1 (**a**) carbon, (**b**) oxygen, (**c**) nitrogen, (**d**) sulfur, (**e**) zinc.

The analysis for pure ZnO showed the elements zinc (Zn) and oxygen (O). Moreover, the gold elements (Au) detected were due to prior Au sputtering, which provided the material’s conductivity in analysis. Furthermore, the most common elements in the samples were O and carbon (C), which is due to the composition of the polymers and the application of the carbon support for the test. Sulfur (S) and nitrogen (N) were identified in GSH-containing samples, which is related to the content of thiol (–SH), amine (–NH_2_), and amide (–CONH–) groups in the protein structure. The successful incorporation of ZnO microparticles was also confirmed by the presence of Zn in sample 40.1 (Figure 5).

### 2.5. Incubation of Materials in Media Simulating Body Fluids 

#### 2.5.1. Potentiometry Analysis

**Figure 6:**
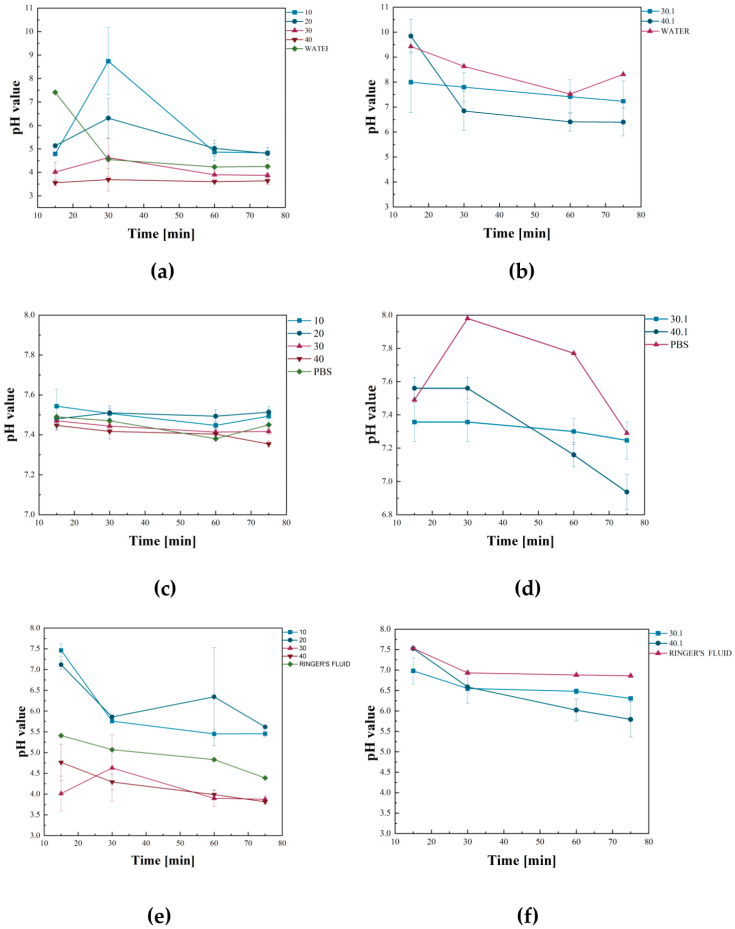
Measured pH values during incubations in (**a**,**b**) distilled water, (**c**,**d**) PBS, (**e**,**f**) Ringer’s fluid.

The potentiometric analysis tracked pH changes, while conductivity measurements evaluated ionic changes in the solutions. Those studies aimed to assess the reactivity and stability of the samples in biological fluid analogs simulating biomedical conditions. During in vitro incubation in liquids simulated body fluids, pH changes in the solutions were measured to determine the effect of the selected chemical composition of the biomaterials on the pH value. The results of the test are shown in Figure 6. What is important is that the impact of added GSH in lowering the pH compared to samples without protein was observed. The acidic nature of the peptide itself causes this. The most significant changes in pH value were identified with distilled water for sample no. 40. The only abrupt change was observed after 30 min for sample no. 20, at which point the value was highest. The changes in pH value may be due to the leaching of the uncrosslinked polymers. In the phosphate-buffered saline (PBS), the incubation process for all samples was stable without sudden changes. Samples containing GSH successively lowered the pH value. Compared to the other solutions, the more minor changes in pH value are due to the buffering character of the PBS. An analogous situation occurred with Ringer’s fluid. Samples containing GSH effectively reduced the pH value of the solution throughout the entire incubation period. A rapid change was noticed for sample 20 after 30 min of incubation. It is related to the reaction of the solution ions with the leaching of the under-crosslinked polymers, as in the case of water. The incubation of the rest of the samples was stable, with no sudden changes. An increase in pH values was also observed for all solutions for the non-GSH samples. In addition, the ZnO content in samples 30.1 and 40.1 does not significantly influence the change in pH value. The decrease in pH occurs as a result of the incorporated GSH. No significant discrepancies in pH values were observed when comparing samples containing 0.5 g of GSH and those containing 1 g. However, these samples effectively lower the pH value, which is highly recommended for treating acne.

#### 2.5.2. Conductivity Analysis

**Figure 7:**
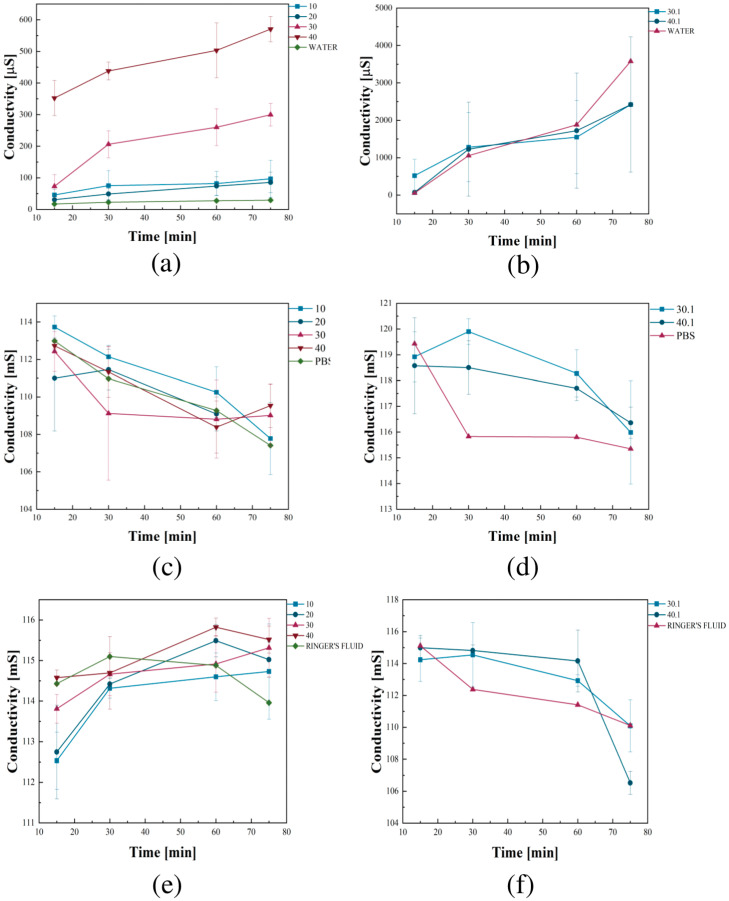
Measured conductivity values during incubation in (**a**,**b**) distilled water; (**c**,**d**) PBS; (**e**,**f**) Ringer’s fluid.

The incubated samples were subjected to an ionic conductivity analysis. This test is based on the changes in ion concentration between the tested solution and the biomaterials. Figure 7 shows the exact results. This test is based on the changes in ion concentration between the solution and the biomaterial.

The most remarkable changes in conductivity were observed for polymeric hydrogels containing GSH incubated in distilled water. Furthermore, the most significant increase in conductivity values was observed for sample no. 40 compared to pure distilled water. This effect results from the release of GSH, which partially dissociates, leading to an increased concentration of ions in the solution. The increase in conductivity occurred stably without abrupt changes. In the case of zinc-added samples, no increase in the conductivity values of the distilled water was observed. A slight decrease in values was noted compared to pure distilled water. The whole incubation process was stable without abrupt changes. The addition of zinc caused a reduction in the dissociation process of GSH. In the case of PBS, minimal changes in conductivity values were observed; however, they were sufficiently small to conclude that the materials were stable in this solution. The same conclusion was reached for Ringer’s fluid. A minimal increase was observed for samples 10–40. A decrease in conductivity was noted for samples with ZnO added. It was stable throughout the incubation period in both cases, coinciding with the corresponding sample of clear Ringer’s solution.

The increase in conductivity occurred stably without abrupt changes. In the ZnO microparticle-added samples, no increase in the conductivity values of the distilled water was observed. The whole incubation process was stable without abrupt changes. Moreover, ZnO microparticles caused a reduction in the dissociation process of GSH. In the case of incubation in PBS, minimal changes in conductivity values were observed; however, they were sufficiently small to conclude that the materials were stable in PBS. The same conclusion was reached for Ringer’s fluid. A minimal increase was observed for samples 10–40. Moreover, a decrease in conductivity was noted for samples with ZnO added. It was stable throughout the incubation period in both cases, coinciding with the corresponding sample of clear Ringer’s fluid.

#### 2.5.3. Swelling Capacity

**Figure 8:**
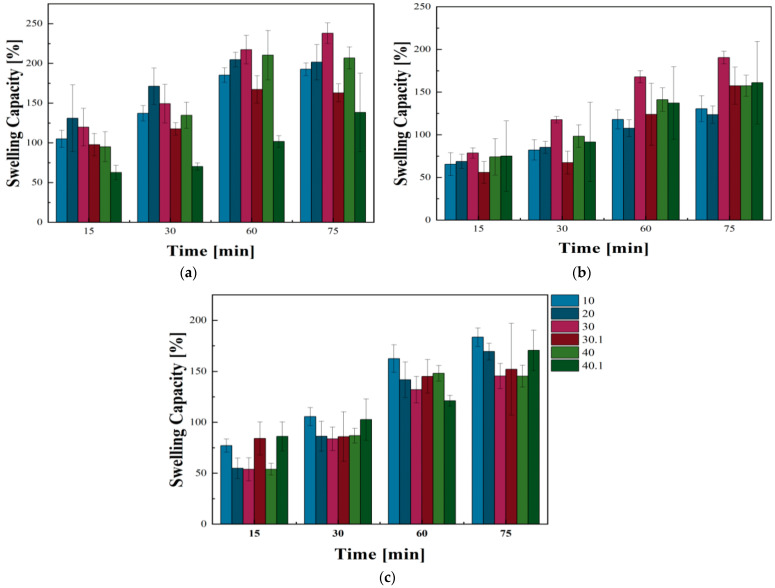
Changes in swelling capacity depending on the medium in which the experiments were conducted. Swelling ability during incubation of polymeric hydrogels in (**a**) distilled water, (**b**) PBS, (**c**) Ringer’s fluid.

To accurately test the swelling capacity of each sample, the polymer hydrogels were incubated in distilled water, Ringer’s liquid, and PBS solution. The results of the swelling test performed on the biomaterials are presented in Figure 8. The swelling capacity of all prepared materials increased with incubation time. In both distilled water and PBS, sample no. 30 showed the highest swelling capacity, which was 190.55 ± 7.48% and 234 ± 12.98% after 75 min for PBS and distilled water, respectively. Furthermore, samples containing ZnO microparticles showed a reduced swelling capacity compared to samples modified with GSH. All GSH-containing hydrogels showed a swelling capacity above 150% in PBS, while sample no. 20 showed the lowest capacity, at 123.63 ± 10.23%. When the hydrogels were incubated in distilled water, samples 20, 30, and 40 showed swelling capacity above 200% after 75 min of the experiment. In turn, samples no. 10 and 30.1 showed swelling capacity below 150%, and sample 40.1 showed the lowest swelling capacity, which was 138.27 ± 49.32%.

Remarkably, the results of tests conducted in Ringer’s fluid significantly differ from those obtained in other media. In Ringer’s fluid, all samples except samples no. 30 and 40 showed swelling capacity above 150%, of which, for sample 10, the capacity was 183.5 ± 9.11%. To summarize, the variations in the swelling capacity of the materials resulting from the measurement solution used result from the chemical composition of the respective fluids. Pure distilled water does not contain ions that can react with the material. PBS has sodium (Na^+^) and chloride ions (Cl^−^), which maintain the isotonicity of the solution. Furthermore, it contains phosphates, which provide the solution with a buffering nature. Besides Na^+^ and Cl^−^ ions, Ringer’s fluid contains calcium (Ca^2+^) and potassium ions (K^+^), giving it the character of a plasma, further influencing the test results.

### 2.6. Indirect Cytotoxicity Test

**Figure 9:**
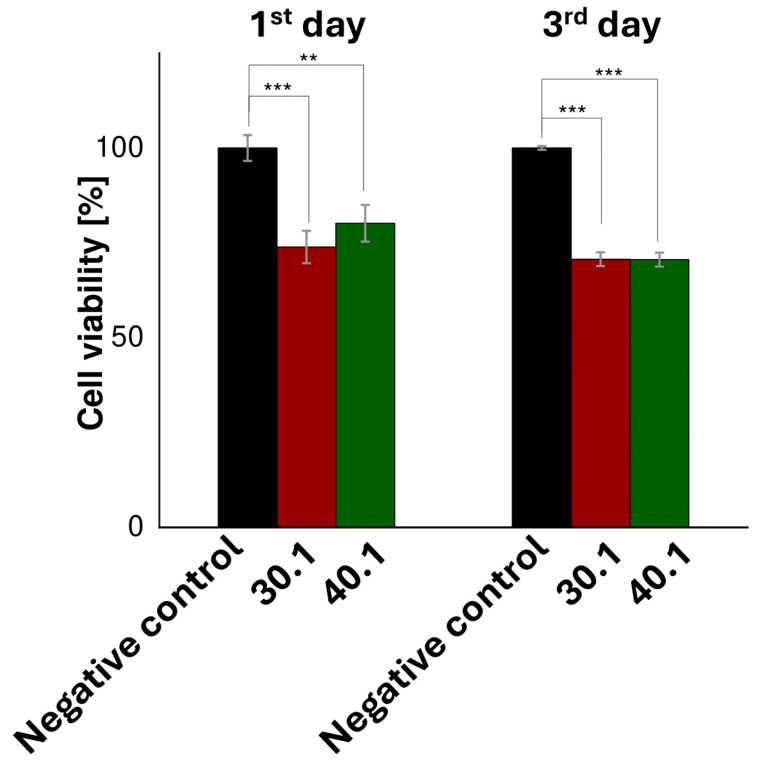
Indirect cytotoxicity of prepared polymeric hydrogels was assessed using a spectrophotometric assay that measures resazurin reduction. Results are considered statistically significant at the following levels: ** *p* < 0.01, and *** *p* < 0.001.

To assess the toxicity of the prepared materials, indirect cytotoxicity tests were conducted, where extracts from materials incubated in cellular media were analyzed. These experiments followed the guidelines set by ISO 10993-5 [37]. After incubating the materials in a culture medium, Dullbecco’s Modified Eagle’s Medium (DMEM) for 24 h, extracts were tested. Mouse fibroblast (L929) cells were exposed to these extracts for another 24 and 72 h, and cytotoxicity was evaluated through resazurin reduction. The results indicated that after 24 and 72 h of exposure to the prepared extracts, cell viability remained above 70% compared to the negative control, which was DMEM. For samples no. 30.1 and 40.1 on the 1st day of the experiment, cell viability is equal to 73.88 ± 4.27% and 80.13 ± 4.84%, respectively. On the 3rd day of the experiment, cell viability for sample no. 30.1 and 40.1 was 70.67 ± 1.79% and 70.56 ± 1.83%, respectively (Figure 9). Common sources of cytotoxicity include residues from organic solvents used in synthesis, intermediate products generated during synthesis, degradation products, and the additives used to modify the polymer [48].

## 3. Discussion

The pathogenesis of acne vulgaris results from a multifaceted process in the hair and sebum region, resulting in bacterial overgrowth and inflammation. Inadequate treatment or complete neglect of treatment can lead to abnormal skin healing, resulting in scarring. To prevent further development of the condition, in addition to a proper diet abstinent from stimulants (i.e., cigarettes) [44], patients should avoid comedogenic products and products that are oil-based [45]. A basic approach to acne treatment is well-chosen skin care. The use of antibacterial properties of the materials used to reduce the presence of *P. acnes* is indicated [46]. However, because bacteria have developed a resistance to antibiotics, there has been an increased interest in other active substances, including nanoparticles and compounds of natural origin with antibacterial properties. A study of ZnO-enriched hydrogel face masks containing GSH provides compelling evidence for their potential use in treating acne vulgaris.

In our work, we demonstrated the possibility of obtaining ZnO microparticles by wet precipitation technique. Analysis of the particle size showed that the structures had a size in the range of 2.371 ± 0.307 µm (Table 1); however, SEM analysis showed a tendency for the particles to agglomerate (Figure 4a). Similar results were shown in a paper described by Bhardwaj et al., where the wet synthesis of ZnO nanoparticles (nZnO) was carried out using ZnSO_4_ and dilute ammonia solution. SEM analysis showed that the materials obtained are flower-like nanostructures, which tend to agglomerate to 1–2 µm structures in size [49]. Furthermore, a study of the preparation of nZnO using wet synthesis and zinc nitrate hexahydrate (Zn(NO_3_)_2_ 6H_2_O) and ammonium hydroxide (NH4OH) as a starting and the addition of polyvinylpyrrolidone (PVP) as a capping agent, presented by Debanath et al., suggests similar conclusions. Despite obtaining nZnO with a size of 8.5 nm, confirmed by XRD analysis, the SEM analysis showed a picture indicating the agglomeration of individual nanoparticles [50].

A critical parameter that must be fulfilled for a biomaterial to act as an active substance carrier is the swelling capacity. The addition of SA effectively increased the swelling index compared to materials prepared using PEG 6000 itself. This is confirmed in a paper published by Iza et al., in which a study of the swelling capacity of PEG 6000 as a function of the molar concentration of polymer to a crosslinking agent, in a distilled water environment, and at a temperature of 25 °C, showed a maximum swelling index of approximately 100% (concentration 1:1) [51]. An increase in swelling capacity after adding SA was also observed in an article on the characterization of nanogels presented by Koehler et al. [52].

In the indirect cytotoxicity test, cell viability decreased after 96 h of incubation to 70.67 ± 1.79% and 70.56 ± 1.83% for samples 30.1 and 40.1, respectively (Figure 9). This decrease could be caused by degradation products, synthesis intermediates, or residual solvents. However, this decrease could also be dictated by the incomplete degradation of GSH, which could have led to a burst release of ZnO. Similar results and conclusions were presented in the work presented by Kwon Oh et al. The authors synthesized nanogels designed to release doxorubicin. After 96 h, the survival of HeLa cells decreased to 60% in the sample where glutathione (0.08 mg/mL) was added after 48 h of incubation to release doxorubicin [53].

## 4. Materials and Methods

### 4.1. Materials

The reactants necessary to obtain ZnO, i.e., sodium hydroxide (NaOH), zinc acetate (Zn(CH_3_COOH)_2_), and the reagents needed to formulate Ringer’s fluid, i.e., NaCl, KCl, and CaCl_2_ were obtained from Chempur (Piekary Śląskie, Poland). Phosphate-buffered saline (PBS) was purchased from Oxoid tablets (Basingstoke, UK). PEG 6000, glutathione (GSH), and poly(ethylene glycol) diacrylate (PEGDA) 700 and 2-hydroxy-2-methylpropionate were kindly provided by Sigma-Aldrich (Darmstadt, Germany). Sodium alginate was bought from Agnex Białystok, Białystok, Poland).

### 4.2. Synthesis of ZnO Microparticles

In order to synthesize zinc oxide microparticles (ZnO), firstly, a 0.3 M NaOH solution was made. Then, 6.57 g of Zn(CH_3_COOH)_2_ was dissolved in distilled water. The Zn(CH_3_COOH)_2_ solution was transferred to a three-necked glass, and the NaOH solution was added while stirring continuously. The mixture was heated to 90 °C with a reflux and maintained under constant stirring for 30 min. The final solution was transferred and rinsed several times with distilled water until the pH value reached neutral. Drying was carried out at 100 °C for 24 h.

### 4.3. Preparation of Biomaterials

To obtain biomaterials, 20 (*w*/*v* %) solutions of PEG 6000 and 2 (*w*/*v* %) of SA were prepared. With continuous stirring, GSH was added to the PEG 6000 solution appropriately. Later, a proper amount of SA was added. All the added amounts and the proportions in which they were distributed in the samples are shown in Table 3. After that, the PEGDA 700 was added to the mixture. Next, the reactants were mixed, and ZnO was added in 0.1 (*w*/*w* %). Then, the mixture was placed in an ultrasonic cleaner for 5 min to dispose of the ZnO microparticle agglomeration. In the final step, a photoinitiator (2-hydroxy-2-methylpropionate) was added, and the mixture was transferred to a 24-well plate and exposed to UV light for 6 min.

### 4.4. Methods

#### 4.4.1. Particular Size

Particle size analysis of the resulting ZnO powder was carried out using an Anton Paar PSA 1190 particle size analyzer (Anton Paar, Graz, Austria). Measurements were made in the presence of ultrasound and were repeated three times.

#### 4.4.2. X-Ray Diffraction (XRD)

For the crystallographic analysis of ZnO, an XRD measurement was performed using a Malvern Panalytical Aeris X-ray diffractometer with a PIXcel1D-Medipix3 detector (Malvern Panalytical, Worcestershire, UK). The measurement was performed with a step of 0.0027° in the 2θ range of 15–80°.

#### 4.4.3. Fourier-Transform Infrared Spectroscopy (FTIR)

A Nicolet iS5 FTIR (Thermo Scientific, Loughborough, UK) spectrophotometer equipped with an iD7 was used. Due to the monolithic diamond crystal, there is a high optical contact between the sample and the diamond, resulting in a clear spectrum. The FT-IR spectra were recorded in the 400 to 4000 cm^−1^ range.

#### 4.4.4. Scanning Electron Microscope (SEM)

A morphological analysis was conducted on the coatings obtained before the incubation period. A TM3000 tabletop scanning electron microscope (SEM) (Hitachi, Tokyo, Japan) equipped with a Quantax 400 V EDS system was used for the analysis. EDS microanalysis was also performed to examine the surface morphology of the coatings and identify any deposits on the sample surfaces. Before SEM analysis, the dried sample surfaces were coated with a thin layer of gold. However, the presence of gold, besides ZnO, was excluded from the study during EDS measurements. EDS microanalysis was performed to determine the elemental composition and generate surface maps of the samples.

#### 4.4.5. Incubation of Materials in Media Simulating Body Fluids

The prepared samples were incubated in vitro in phosphate-buffered saline (PBS), Ringer’s fluid, and distilled water, whose compositions are shown in Table 4, at room temperature for 1 h. Each sample was tested three times. Measurements were taken for 15, 30, 60, and 75 min.

##### Potentiometry

For potentiometric analysis, biomaterial samples were incubated in PBS, Ringer’s fluid, and distilled water under controlled conditions at room temperature for 1 h. pH measurements were taken at specified intervals (15, 30, 60, and 75 min) using a pH meter equipped with the electrode.

##### Conductivity

Conductivity measurements were performed to assess changes in ion concentrations in the incubation solutions. The biomaterial samples were placed in sterile containers filled with 30 mL of PBS, Ringer’s fluid, or distilled water and incubated at room temperature for 1 h. Measurements were taken using a conductivity meter for 15, 30, 60, and 75 min.

##### Swelling Capacity

Biomaterial samples were placed in sterile containers containing 30 mL of PBS, Ringer’s fluid, or distilled water to assess swelling capacity. The incubation was conducted at room temperature for 1 h. After the specified time, the samples were removed, lightly drained on filter paper to remove excess liquid, and weighed. The swelling capacity was calculated using the formula:

$$\mathrm{Swelling}\;\mathrm{Capacity}=\frac{m1-m0}{m0}\times 100\%,$$

where m1 is the mass of the sample after incubation; m0 is the dry mass of the sample.

#### 4.4.6. Cell Culture Conditions for Mouse Fibroblast (L929)

The L929 cell line was maintained in Dulbecco’s Modified Eagle Medium (DMEM) with a glucose concentration of 4500 mg/L. The culture medium was supplemented with 10% (*v*/*v*) fetal bovine serum (FBS), 2.5 mM L-glutamine, 10 µg/mL penicillin, 100 µg/mL streptomycin, and 0.625 µg mL^−1^ amphotericin B. Cultures were incubated at 37 °C in a humidified atmosphere containing 5% CO_2_. Experimental procedures were initiated when the cells reached a minimum confluence of 85%.

For cell passage, the culture medium was carefully discarded, and the adherent cells were rinsed with Dulbecco’s phosphate-buffered saline (DPBS). Subsequently, the cells were incubated sequentially with 0.25% trypsin solution for 5 min. After incubation, the cells were resuspended in a fresh culture medium and subjected to centrifugation at 1000 rpm for 5 min. The resultant cell pellet was then resuspended in an appropriate volume of culture medium. Cell viability and count were determined using an automatic hemocytometer after staining with trypan blue dye, differentiating viable cells from non-viable cells.

#### 4.4.7. Indirect Cytotoxicity Test

The experiments were conducted in accordance with the ISO 10993-5:2009 standard [37]. Extracts were prepared by incubating patches of DMEM for 24 h. Specifically, 100 mg of the relevant biomaterial was incubated in 1 mL of DMEM at 37 °C within a 5% CO_2_ atmosphere for 24 h, aligned with ISO 10993-12 guidelines [54]. Concurrently, L929 cells were seeded into 96-well plates at a density of 2 × 10^4^ cells per well. These plates received 200 µL of medium and were incubated for 24 h under the same conditions of 37 °C and 5% CO_2_. After the initial incubation, the DMEM was aspirated, and an equivalent volume of the prepared extracts (200 µL) was introduced to each well. The plates were then re-incubated for an additional 24 and 72 h under identical conditions. Wells containing DMEM served as negative controls throughout the experimental procedure.

The extracts and DMEM were removed from the wells, and 200 µL of a 100 µM resazurin solution was added. The plates were incubated in the dark for 4 h at 37 °C with 5% CO_2_. After incubation, 150 µL of each solution was transferred to a new 96-well plate. The absorbance was then measured at wavelength of 570 and 630 nm using a FLX800 plate reader (BIO-TEK Instruments, Winooski, VT, USA). The results were expressed as a percentage of cell viability relative to the negative control.

### 4.5. Statistical Analysis

The results are shown as mean values along with their standard errors (SE). We used an analysis of variance (ANOVA) to compare the two populations in groups. Significant differences were identified at the following probability levels: * *p* < 0.05, ** *p* < 0.01, and *** *p* < 0.001, using Origin Pro 2019 Software.

## 5. Conclusions

From our study, it can be concluded that GSH-modified hydrogel face masks containing ZnO microparticles may find application in treating skin disorders, including those caused by acne vulgaris. XRD, FT-IR, and EDS analyses confirmed the validity of the zinc synthesis. Otherwise, EDS and FT-IR analyses identified the elements in the biomaterials. In vitro incubation studies confirmed interactions of the polymeric hydrogels with body fluids, including changes in pH and conductivity, suggesting active ion exchange. The observed pH changes toward acidic values were related to the presence of GSH, while ZnO showed no significant effect on this parameter. The obtained biomaterials have high swelling capacity (>100%), which was depending on the medium, with the highest absorption for sample no. 30, for distilled water and PBS, indicating the effectiveness of the coating modification. A cytotoxicity test carried out in accordance with ISO 10993-5 showed that the materials obtained were not cytotoxic.

The next research steps should focus on performing in-depth analysis to demonstrate the biomedical potential of the synthesized materials. Such tests include, but are not limited to, indirect proliferation tests, determination of the amount of collagen secreted, confirmation of anti-inflammatory potential. Furthermore, it is worth considering experiments to determine the oxidative stress in contact with the prepared biomaterials. From a materials point of view, future work should focus on obtaining materials in a way that reduces the tendency to agglomerate ZnO microparticles.

## Figures and Tables

**Table 1:** Size distribution of synthesized ZnO particles.

Number	Mean Particle Size [µm]	D_10_ [µm]	D_50_ [µm]	D_90_ [µm]
Mean value	2.371 ± 0.307	0.594 ± 1.114	1.514 ± 0.080	4.780 ± 0.014

**Table 2:** Elemental composition of the tested coatings and pure ZnO.

Sample	Mass [%]
10	C: 57.48; O: 42.52
20	C: 58.04; O: 41.96
40	C: 46.72; O: 50.37; N: 2.01; S: 0.9
40.1	C: 46.04; O: 47.47; N: 3.23; S: 1.79; Zn:1.48
ZnO	C: 69.69; O: 20.44; Zn: 8.28; Au: 1.59

**Table 3:** Composition of the biomaterials.

Name of the Sample	Abbreviation	PEG 6000 [mL]	SA [mL]	PEGDA 700 [mL]	GSH [g]	ZnO [*w*/*w*%]	Photoinitiator [µL]
PEG	10	5	-	1.3	-	-	28
PEG/SA	20		2		-	-	
PEG/SA/0.5GSH	30				0.5	-	
PEG/SA/0.5GSH/ZnO	30.1				0.5	0.1	
PEG/SA/1GSH	40				1.0	-	
PEG/SA/1GSH/ZnO	40.1				1.0	0.1	

**Table 4:** Composition of simulated biological fluids.

Component [g/L]	PBS	Ringer’s Fluid
NaCl	8.000	8.600
KCl	0.200	0.300
CaCl_2_	-	0.423
Na_2_HPO_4_	1.150	-
KH_2_PO_4_	0.200	-

## Data Availability

Data that support the findings of this study are contained within the article.

## Abbreviations

10	Sample containing PEG 6000, PEGDA 700, and photoinitiator
20	Sample containing PEG 6000, PEGDA 700, SA, and photoinitiator
30	Sample containing PEG 6000, PEGDA 700, SA, 0.5 [g] GSH and photoinitiator
30.1	Sample containing PEG 6000, PEGDA 700, SA, 0.5 [g] GSH, ZnO, and photoinitiator
40	Sample containing PEG 6000, PEGDA 700, SA, 1 [g] GSH, and photoinitiator
40.1	Sample containing PEG 6000, PEGDA 700, SA, 1 [g] GSH, ZnO, and photoinitiator
D_10_	Particle size distribution of 10% of the total volume
D_50_	Particle size distribution of 50% of the total volume
D_90_	Particle size distribution of 90% of the total volume
D_mean_	mean diameter of the particle’s size
DMEM	Dullbecco’s Modified Eagle’s Medium
EDS	Energy Dispersive Spectroscopy
FT-IR	Fourier-Transform Infrared Spectroscopy
GSH	Glutathione
GPx	Glutathione peroxidase
L929	Mouse fibroblast cell line
PBS	Phosphate-buffered saline
PEG 6000	Polyethylene glycol
PEGDA	Poly(ethylene glycol) diacrylate
Photoinitiator	2-hydroxy-2-methylpropionate
ROS	Reactive oxygen species
SA	Sodium alginate
SEM	Scanning Electron Microscopy
XRD	X-ray Diffraction
ZnO	Zinc oxide
